# Author Correction: In situ imaging reveals disparity between prostaglandin localization and abundance of prostaglandin synthases

**DOI:** 10.1038/s42003-021-02560-w

**Published:** 2021-08-20

**Authors:** Kyle D. Duncan, Xiaofei Sun, Erin S. Baker, Sudhansu K. Dey, Ingela Lanekoff

**Affiliations:** 1 Department of Chemistry-BMC, Uppsala University, Uppsala, Sweden; 2 Division of Reproductive Sciences, Cincinnati Children’s Hospital Medical Center, Cincinnati, OH 45229 USA; 3 College of Medicine, University of Cincinnati, Cincinnati, OH 45221 USA; 4 Department of Chemistry, North Carolina State University, Raleigh, NC USA

**Keywords:** Biosynthesis, Molecular biology

Correction to: *Communications Biology* 10.1038/s42003-021-02488-1, published online 13 August 2021.

The original version of this Article contained an error in the author affiliations.

The affiliation of Xiaofei Sun and S. K. Dey with “Division of Reproductive Sciences, Cincinnati Children’s Hospital Medical Center, Cincinnati, OH 45229, USA” was inadvertently omitted.

This has now been corrected in both the PDF and HTML versions of the Article.

