# Supplementary material for: In situ imaging reveals disparity between prostaglandin localization and abundance of prostaglandin synthases
Source: Commun Biol. 2021 Aug 13;4:966. doi: 10.1038/s42003-021-02488-1 (PMC8363604; doi:10.1038/s42003-021-02488-1)
Supplement: Supplementary file 2 — Supplementary Information [file 42003_2021_2488_MOESM2_ESM.pdf]

Supplementary Materials

In situ imaging reveals disparity between prostaglandin localization and abundance of prostaglandin synthases

Kyle D. Duncan[1+], Xiaofei Sun[2+], Erin Baker[3], Sudhansu K. Dey[2], and Ingela Lanekoff[1*]

1) Department of Chemistry-BMC, Uppsala University, Uppsala, Sweden,
2) Division of Reproductive Sciences, Cincinnati Children's Hospital Medical Center, Cincinnati, OH 45229, USA,
3) Department of Chemistry, North Carolina State University, Raleigh, NC 27695, USA

+Co-first authors
*Corresponding author

Corresponding author:

Dr. Ingela Lanekoff
Ingela.Lanekoff@kemi.uu.se
Dept. of Chemistry-BMC (599)
Uppsala University
751 24 Uppsala
Sweden

Table S1. RT primers used for segmented RT-qPCR (Figure 2, Figure 3)

**Name**	**Sequence**
Hpgds-F	tcgccttctgaaagatttgg
Hpgds-R	aaaccaaaagtgtggtgctg
Ptgds-F	agtgcagcccaactttcaac
Ptgds-R	ggctaccactgtcttgcaca
Ptgdr-F	tggctctcatgacagtgctc
Ptgdr-R	caaggcttggaggtcttctg
Ptgdr2- F	ctggtctcaaccaatcagca
Ptgdr2-R	gtcacccaggaaccagaaga
Rpl7-F	gcagatgtaccgcactgagattc
Rpl7-R	acctttgggcttactccattgata
Pgdh-F	tggctgaccagaaacaactg
Pgdh-R	ccgcttttcatgaggttagc
Ptgs2-F	ccacttcaagggagtctgga
Ptgs2-R	gagaaggcttcccagctttt
Ptgs1-F	aggagatggctgctgagttgg
Ptgs1-R	aatctgactttctgagttgcc

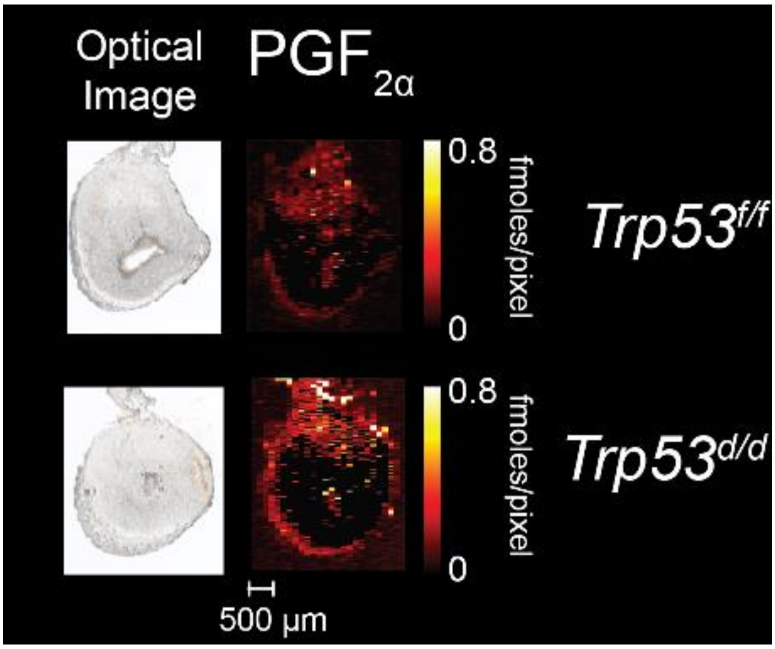

Figure S1. Mass spectrometry images for $PGF_{2\alpha}$ from *Trp53*$^{f/f}$ and *Trp53*$^{d/d}$ mice.

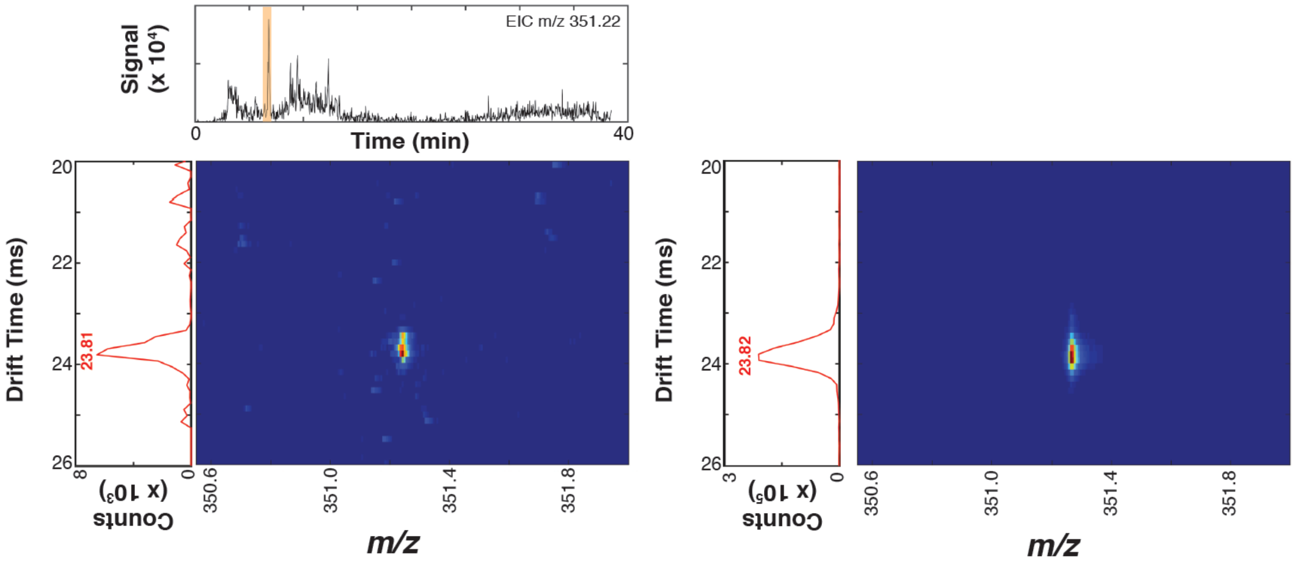

Figure S2. Ion mobility data plots for tissue (left) and PGD_2 standard (right)

Table S2. Drift times for individual standards of $[M-H]^-$ PG isomers at *m/z* 351.217

Isomer	Drift time
PGD_2	23.82
PGE_2	23.98
PGH_2	23.92
PGI_2	24.15
LXA_4	24.28

Table S3. Drift times for PGD_2 in *Trp53*$^{f/f}$ and *Trp53*$^{d/d}$ samples

		**Drift time**	**Average**
***Trp53*$^{f/f}$**	1	23.81	
	2	23.76	
	3	23.79	
	4	23.76	
	5	23.78	
	6	23.75	**23.78**
***Trp53*$^{d/d}$**	1	23.79	
	2	23.81	
	3	23.81	
	4	23.79	
	5	23.79	
	6	23.81	**23.80**

Table S4. Mass list of targeted PG and PG precursor metabolites detected with nano-DESI MSI

Name	Abbreviation	Chemical formula	Accurate mass (Da)	$[M+^{107}AG]^+$ accurate mass (Da)
2-Arachidonoylglycerol	2-AG	$C_{23}H_{38}O_4$	378.2270	485.1821
Arachadonic acid	AA	$C_{20}H_{32}O_2$	304.2402	411.1453
Prostaglandin D_2	PGD_2	$C_{20}H_{32}O_5$	352.2250	459.1300
Prostaglandin $F_{2\alpha}$	$PGF_{2\alpha}$	$C_{20}H_{34}O_5$	354.2406	461.1457
Prostaglandin $F_{2\alpha}$-d_9	$PGF_{2\alpha}$-d_9	$C_{20}H_{25}D_9O_5$	363.3111	470.2162

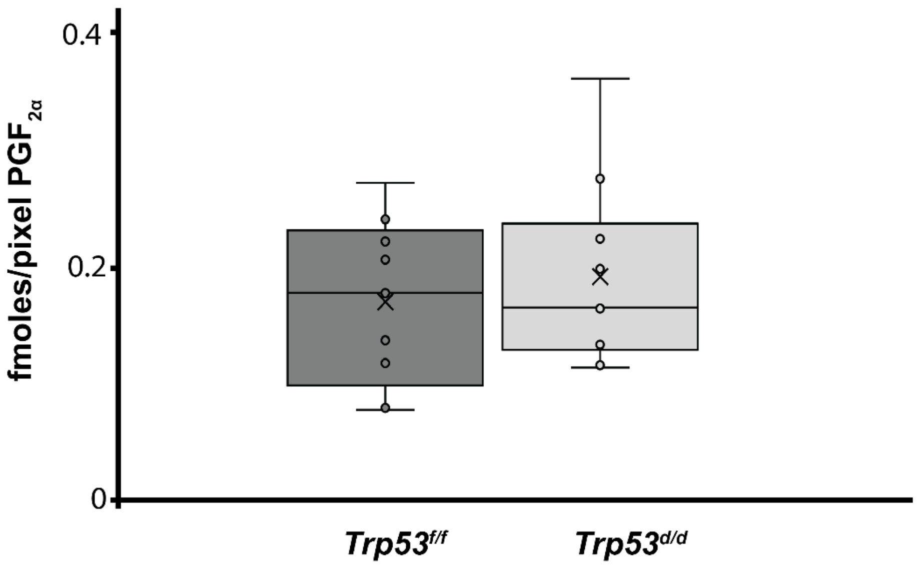

Figure S3. Regions of interest for the myometrium of *Trp53*$^{f/f}$ and *Trp53*$^{d/d}$ mice with detected concentrations of $PGF_{2\alpha}$ from 3 biological and 3 technical replicates (n=9 for *Trp53*$^{f/f}$ and n=10 for *Trp53*$^{d/d}$).

Table S5: Raw data for quantitative mass spectrometry imaging of *Trp53*$^{f/f}$ implantation sites.

	Trp53$^{f/f}$		
	**2-AG**	**AA**	**PGD_2**
n	**M pole (fmoles/pixel)**		
1	0.91	4.83	0.48
2	0.54	3.50	0.41
3	0.44	3.68	0.35
4	0.43	2.72	0.16
5	0.68	2.78	0.20
6	0.71	3.88	0.29
7	0.86	4.14	0.27
8	1.98	6.67	0.35
9	0.89	5.41	0.43
**mean**	**0.83**	**4.18**	**0.33**
n	**AM pole (fmoles/pixel)**		
1	1.61	9.66	0.62
2	1.03	7.12	0.43
3	0.87	9.35	0.63
4	0.91	6.71	0.27
5	1.39	7.06	0.30
6	1.58	10.38	0.51
7	1.32	9.88	0.68
8	2.42	15.78	1.00
9	1.85	13.06	0.82
**mean**	**1.44**	**9.89**	**0.58**

Note: technical replicate images 1-3 are from mouse 1, 3-5 are from mouse 2, and 7-9 are from mouse 3

Table S6: Raw data for quantitative mass spectrometry imaging of *Trp53*$^{d/d}$ implantation sites.

	Trp53$^{d/d}$		
	**2-AG**	**AA**	**PGD_2**
n	**M pole (fmoles/pixel)**		
1	0.56	1.93	0.22
2	3.72	7.63	0.53
3	0.81	3.98	0.26
4	0.40	2.77	0.29
5	0.65	2.85	0.30
6	0.58	2.05	0.22
7	1.00	4.94	0.43
8	1.11	4.65	0.40
9	1.66	7.29	0.65
10	1.49	6.95	0.85
**mean**	**1.20**	**4.50**	**0.42**
n	**AM pole (fmoles/pixel)**		
1	1.97	7.67	0.53
2	4.11	16.52	1.23
3	1.90	10.28	0.48
4	1.25	13.43	0.99
5	2.04	9.66	0.55
6	2.61	10.20	0.56
7	3.92	19.68	1.17
8	3.49	16.12	0.82
9	5.43	24.18	1.99
10	4.49	21.26	1.26
**mean**	**3.12**	**14.90**	**0.96**

Note: technical replicate images 1-3 are from mouse 1, 3-5 are from mouse 2, and 7-10 are from mouse 3
