# Supplementary material for: In situ imaging reveals disparity between prostaglandin localization and abundance of prostaglandin synthases
Source: Commun Biol. 2021 Aug 13;4:966. doi: 10.1038/s42003-021-02488-1 (PMC8363604; doi:10.1038/s42003-021-02488-1)
Supplement: Supplementary file 3 — Description of Supplementary Files [file 42003_2021_2488_MOESM3_ESM.pdf]

Description of Additional Supplementary Files

File name: Supplementary Data 1

Description: Source data used to generate Figures.
