# Supplementary material for: In situ imaging reveals disparity between prostaglandin localization and abundance of prostaglandin synthases
Source: Commun Biol. 2021 Aug 13;4:966. doi: 10.1038/s42003-021-02488-1 (PMC8363604; doi:10.1038/s42003-021-02488-1)
Supplement: Supplementary file 5 — Reporting Summary [file 42003_2021_2488_MOESM5_ESM.pdf]

nature portfolio

Corresponding author(s): Ingela Lanekoff

Last updated by author(s): Jul 15, 2021

Reporting Summary

Nature Portfolio wishes to improve the reproducibility of the work that we publish. This form provides structure for consistency and transparency in reporting. For further information on Nature Portfolio policies, see our Editorial Policies and the Editorial Policy Checklist.

Statistics

For all statistical analyses, confirm that the following items are present in the figure legend, table legend, main text, or Methods section.

n/a	Confirmed	
☐	☒	The exact sample size (*n*) for each experimental group/condition, given as a discrete number and unit of measurement
☐	☒	A statement on whether measurements were taken from distinct samples or whether the same sample was measured repeatedly
☐	☒	The statistical test(s) used AND whether they are one- or two-sided<br>*Only common tests should be described solely by name; describe more complex techniques in the Methods section.*
☒	☐	A description of all covariates tested
☒	☐	A description of any assumptions or corrections, such as tests of normality and adjustment for multiple comparisons
☐	☒	A full description of the statistical parameters including central tendency (e.g. means) or other basic estimates (e.g. regression coefficient) AND variation (e.g. standard deviation) or associated estimates of uncertainty (e.g. confidence intervals)
☐	☒	For null hypothesis testing, the test statistic (e.g. *F*, *t*, *r*) with confidence intervals, effect sizes, degrees of freedom and *P* value noted<br>*Give P values as exact values whenever suitable.*
☒	☐	For Bayesian analysis, information on the choice of priors and Markov chain Monte Carlo settings
☒	☐	For hierarchical and complex designs, identification of the appropriate level for tests and full reporting of outcomes
☒	☐	Estimates of effect sizes (e.g. Cohen's *d*, Pearson's *r*), indicating how they were calculated

Our web collection on statistics for biologists contains articles on many of the points above.

Software and code

Policy information about availability of computer code

Data collection	Mass Spectrometry Imaging: Xcaliber software from ThermoFisher Scientific<br>LC-IMS-MS - Agilent mass hunter
Data analysis	Mass spectrometry Imaging: Matlab 2019b, simple functions to generate image matrices and perform statistical analysis are available upon request. LC-IMS-MS - Agilent MassHunter IM-MS Browser, qPCR - Graphpad

For manuscripts utilizing custom algorithms or software that are central to the research but not yet described in published literature, software must be made available to editors and reviewers. We strongly encourage code deposition in a community repository (e.g. GitHub). See the Nature Portfolio guidelines for submitting code & software for further information.

Data

Policy information about availability of data

All manuscripts must include a data availability statement. This statement should provide the following information, where applicable:

- Accession codes, unique identifiers, or web links for publicly available datasets
- A description of any restrictions on data availability
- For clinical datasets or third party data, please ensure that the statement adheres to our policy

All data needed to evaluate the conclusions in the paper are present in the paper and/or the Supplementary Materials. Source data used to generate the figures are available in the Supplementary Data 1 Excel file. Any remaining information can be obtained from the corresponding author upon reasonable request.

Field-specific reporting

Please select the one below that is the best fit for your research. If you are not sure, read the appropriate sections before making your selection.

- [x] Life sciences
- [ ] Behavioural & social sciences
- [ ] Ecological, evolutionary & environmental sciences

For a reference copy of the document with all sections, see nature.com/documents/nr-reporting-summary-flat.pdf

Life sciences study design

All studies must disclose on these points even when the disclosure is negative.

Sample size	The sample size was determined by the minimum number of animal sacrifices required to enable robust statistical comparisons across mouse genotypes. For mass spectrometry imaging experiments, at least three technical replicates were carried out for three biological replicates of each mouse genotype. Therefore, a total of nine mass spectrometry imaging experiments were included for each genotype. All sample sizes for qPCR are indicated on bars or in figure legends in the manuscript.
Data exclusions	Aside from the 18 mass spectrometry images included in our findings, 9 for Trp53f/f and 9 for Trp53d/d mice, several images were discarded due to experimental sources of error. These include specific errors such as disruption to liquid extraction due to clogging nano-DESI capillaries, impinging capillaries onto the tissue surface, or severe bumps and uneven tissue surfaces. All mass spectrometry images of adequate technical quality were included in the data analysis.
Replication	Mass spectrometry imaging experiments included 3 technical replicate images for reach biological replicate (mouse) for both Trp53f/f and Trp53d/d mice to ensure statistical robustness.
Randomization	Mass spectrometry imaging experiments, approximately 4 hours per image, were carried out in a random order with Trp53d/d and Trp53f/f tissue sections. Additionally, images for each biological replicate animal were randomized to ensure negligible sampling artifacts and biases.
Blinding	Blinding was not possible or relevant for this study as the mouse uterus sections analyzed for Trp53f/f and Trp53d/d mice are morphologically different on Day 8, and therefore recognizable by the investigators upon analysis.

Reporting for specific materials, systems and methods

We require information from authors about some types of materials, experimental systems and methods used in many studies. Here, indicate whether each material, system or method listed is relevant to your study. If you are not sure if a list item applies to your research, read the appropriate section before selecting a response.

Materials & experimental systems

n/a	Involved in the study	
☐	☒	Antibodies
☒	☐	Eukaryotic cell lines
☒	☐	Palaeontology and archaeology
☐	☒	Animals and other organisms
☒	☐	Human research participants
☒	☐	Clinical data
☒	☐	Dual use research of concern

Methods

n/a	Involved in the study	
☒	☐	ChIP-seq
☒	☐	Flow cytometry
☒	☐	MRI-based neuroimaging

Antibodies

Antibodies used	cox2 antibody, cytokeratin 8 antibody
Validation	The Cox2 antibody is validated by Cox2-/- tissues. The cytokeratin 8 (CK8) antibody is a monoclonal antibody sold by 'developmental studies hybridoma bank' has been widely used in the research.

Animals and other organisms

Policy information about studies involving animals; ARRIVE guidelines recommended for reporting animal research

Laboratory animals	mouse P53 floxed mice with a Cre gene driven by progesterone receptor promoters.
Wild animals	No.
Field-collected samples	No.

Ethics oversight	All animals and surgical procedures were approved by IACUC with protocol number IACUC2019-0067<br>All experiments were approved by the Institutional Biosafety Committee: IBC2019-0086<br>All experiments conducted the NIH funding are approved by sponsored Research Program.

Note that full information on the approval of the study protocol must also be provided in the manuscript.
